# Directional Asymmetry Without Timescale Dependence: Longitudinal Associations Between Procrastination and Depressive Symptoms

**DOI:** 10.1155/da/3056943

**Published:** 2026-04-29

**Authors:** Rong Zhang, Huaxian Tan, Lin Wang, Shunmin Zhang

**Affiliations:** ^1^ School of Mental Health and Psychological Sciences, Anhui Medical University, Hefei, China, ahmu.edu.cn; ^2^ Department of Psychology and Behavioral Sciences, Zhejiang University, Hangzhou, China, zju.edu.cn; ^3^ Zhejiang Key Laboratory of Neurocognitive Development and Mental Health, Zhejiang University, Hangzhou, China, zju.edu.cn

**Keywords:** depression, emotion-regulation account, procrastination, timescale

## Abstract

**Background:**

Longitudinal research has examined procrastination‐emotion dynamics across diverse temporal intervals, yet it remains unclear whether findings obtained at different timescales reflect distinct underlying mechanisms. Guided by an emotion‐regulation account, the present study tested the hypotheses that negative emotions predicted subsequent procrastination more strongly at shorter intervals, whereas procrastination predicted increases in depressive symptoms over longer intervals.

**Methods:**

To test whether the procrastination‐depressive symptoms coupling varied across temporal scales, we integrated three longitudinal datasets (i.e., daily, 1‐month, and half‐yearly intervals) and compared models in which within‐person lagged effects (i.e., from depressive symptoms to procrastination and the reverse path) were allowed to vary versus constrained to be invariant across timescales. Directional paths were then reported based on the better‐fitting model.

**Results:**

Model comparisons favored the timescale‐invariant model over the timescale‐variant model for both directional paths. Across multiple temporal scales, the timescale‐invariant model showed negligible effects of depressive symptoms on subsequent procrastination, while procrastination predicted increases in depressive symptoms.

**Conclusion:**

Contrary to timescale‐dependent expectations, no evidence for temporally distinct mechanisms was observed within the examined intervals. Instead, the findings reveal a modest directional asymmetry across timescales, suggesting that procrastination may have consistent but small effects on subsequent increases in depressive symptoms rather than forming a reciprocal feedback loop.

## 1. Introduction

Procrastination is a voluntary delay of an intended course of action despite expecting to be worse off for the delay [1]. Researchers typically conceptualized it either as a fast‐varying behavior in specific tasks (e.g., postponing a particular assignment) or as a slow‐moving tendency across broader task domains [2, 3]. This fast‐slow distinction highlights the temporal heterogeneity of procrastination dynamics, even though the two timescales have rarely been explicitly differentiated in prior empirical work.

A growing body of longitudinal research has documented robust associations between procrastination and negative emotions such as depression and anxiety. Based on the temporal focus of prior studies, these emotion‐procrastination associations can be conceptually classified into two types. Studies examining task‐specific procrastination over short observation windows (e.g., days or weeks) implicitly capture short‐term within‐person couplings between fast‐varying procrastination and momentary negative emotions [4]. In contrast, studies tracking general procrastination tendencies across longer intervals (e.g., months or years) capture long‐term within‐person couplings between slow‐moving changes in procrastination and more enduring emotional distress [5–8]. However, it remains unclear whether these short‐term and long‐term couplings reflect a shared within‐person mechanism operating at different timescales or distinct psychological processes.

An emotion‐regulation account suggests that short‐term and long‐term emotion‐procrastination couplings may be governed by distinct mechanisms. From this perspective, procrastination functions as a maladaptive emotion‐regulation strategy that provides immediate emotional relief but ultimately leads to more emotional distress [9]. At the short‐term timescale, negative emotions may trigger task‐specific procrastination as a means of avoidance, and disengagement from the task can temporarily reduce distress, forming a short‐term within‐person coupling driven by emotion‐regulation processes [10]. Consistent with this view, aversive tasks are associated with a higher frequency of procrastination [11], and mental simulation of delayed task engagement reduces negative emotions relative to simulating immediate engagement [12].

By contrast, the adverse consequences of procrastination are typically realized after extended delays rather than occurring immediately upon task disengagement. These consequences include both self‐evaluative outcomes, such as guilt or self‐blame [13], and situational setbacks, such as accumulated workload, approaching deadlines, or task failures [14, 15]. Although both types of consequences are temporally delayed relative to immediate emotion‐regulation processes, self‐evaluative outcomes are likely to emerge earlier than situational setbacks, as they may arise once individuals recognize the potential consequences of procrastination, whereas situational setbacks typically require those consequences to materialize. Because these consequences emerge gradually and often accumulate over time, they possibly contribute to sustained increases in negative emotions. This process gives rise to long‐term within‐person couplings, in which slow‐moving changes in general procrastination tendencies predict enduring emotional distress (hereafter referred to as the worse‐off effect). Taken together, emotion‐regulation processes are more likely to underlie short‐term couplings, whereas the worse‐off effect characterizes long‐term couplings, with procrastination more strongly predicting subsequent emotional distress at longer timescales.

In contrast to emotion‐regulation processes that may differentially govern emotion‐procrastination couplings across short‐term and long‐term timescales, self‐efficacy may function as a common mechanism across timescales. At the short‐term level, temporal motivation theory suggests that task‐specific self‐efficacy shapes immediate emotional and motivational responses to a given task. Individuals with low self‐efficacy anticipate a low probability of success and a high risk of failure, which amplifies distress (e.g., anxiety and frustration) and reduces intention to engage, thereby increasing fast‐varying, task‐specific procrastination [16].

At the long‐term level, low general self‐efficacy may similarly undermine engagement across a broad range of tasks and increase vulnerability to enduring emotional distress, such as depression and anxiety [17, 18]. Through this pathway, self‐efficacy may contribute to slow‐moving changes in both general procrastination tendencies and emotional distress over time. Taken together, unlike emotion‐regulation and worse‐off processes that are temporally distinct, self‐efficacy is likely to show similar associations with emotional distress and procrastination across short‐term and long‐term within‐person couplings.

Although previous studies have not directly tested the distinctions outlined above, their findings provide converging evidence supporting the plausibility of these distinctions. Specifically, cross‐lagged patterns consistent with short‐term emotion‐procrastination couplings have been reported primarily in studies focusing on task‐specific, short‐term processes, with negative emotions predicting subsequent procrastination but the reverse association often being nonsignificant [4, 15]. In contrast, cross‐lagged patterns consistent with the worse‐off effect have been observed more frequently in studies relying on longer‐term, general measures of procrastination, in which procrastination predicted subsequent distress without reliable reciprocal effects [5–8]. Notably, this timescale‐dependent variation may not extend to constructs like self‐efficacy. Measures conceptually linked to self‐efficacy have shown robust negative cross‐lagged effects on procrastination across both short‐term, task‐focused designs and longer‐term assessments of general procrastination tendencies [19]. Taken together, these patterns do not establish causal mechanisms but suggest that prior longitudinal findings systematically align with a distinction between short‐term and long‐term within‐person emotion‐procrastination couplings, as well as with differential roles of emotion regulation, worse‐off processes, and self‐efficacy.

The present research was originally designed to examine longitudinal associations between procrastination, depression, anxiety, and self‐efficacy within a multitimescale framework. The preregistered studies were conducted using complementary longitudinal designs. Specifically, data were collected using a 14‐day intensive longitudinal design and two three‐wave panel designs with 1‐month and half‐yearly intervals, respectively.

Current work instead focused on probing whether the temporal associations between procrastination and depressive symptoms varied across multiple timescales by integrating data from the complementary designs. This key adjustment was necessitated by a post hoc observation. Specifically, measures of self‐efficacy, depression, and anxiety did not demonstrate adequate longitudinal measurement invariance across waves, which precluded a comprehensive test of the preregistered hypotheses at the construct level. To address this limitation while maintaining methodological rigor, we adopted a more focused analytic strategy by probing the temporal associations between procrastination and depressive symptoms at the item level. This approach not only provided a unified assessment of emotion‐procrastination dynamics across short‐term and long‐term intervals but also avoided assumptions of stable latent factor structures and permitted valid examination of within‐person change over time. Together, the present study hypothesized that depressive symptoms would more strongly predict subsequent procrastination at shorter time intervals, whereas procrastination would predict depressive symptoms across longer time intervals.

## 2. Methods

### 2.1. Designs and Participants

To probe the longitudinal associations among variables, we integrated data from three complementary designs spanning multiple timescales, including an intensive longitudinal design over 14 days and two three‐wave longitudinal designs with 1‐month and half‐yearly intervals. Surveys were preregistered at the Open Science Framework (https://osf.io/qtf36/overview?view_only=9843d03c20e04a7f8dff0a51568748bd), (https://osf.io/ny3ga/?view_only=45b46c64669241c68d44ae89119477b3) and (https://osf.io/xchdg/?view_only=41b0b0fe4a3143fbbecccac58b371711), respectively.

A total of 2730 college students were enrolled at baseline via campus advertisements, with some attrition occurring in subsequent waves. Specifically, Sample 1 comprised 167 participants, none of whom dropped out. Sample 2 collected 698, 473, and 444 questionnaires across waves, respectively. The attrition rate for each variable remained consistent across waves, with rates of 32.23% between wave 1 and wave 2, and 6.13% between wave 2 and wave 3. Sample 3 collected 1865, 1282, and 1051 questionnaires across waves, respectively. The attrition rates for variables were 31.26% between wave 1 and wave 2, and 18.02% between wave 2 and wave 3.

After data screening, a total of 2490 valid participants (i.e., number of subjects at wave 1) were included in the final analyses (see preprocessing of data for details on screening criteria). In detail, Sample 1 consisted of 167 subjects (83 males, M age = 20.53). Sample 2 included 641 participants at wave 1 (response rate: 91.83%; 240 males, M age = 20.15), 424 participants at wave 2 (response rate: 89.64%), and 340 participants at wave 3 (response rate: 76.58%). Sample 3 comprised 1682 valid participants at wave 1 (response rate: 90.19%; 675 males, M age = 18.41), 1077 at wave 2 (response rate: 84.01%), and 959 at wave 3 (response rate: 91.25%).

Overall, attrition analyses indicated nonrandom attrition in Samples 2 and 3. Specifically, in Sample 2, completers and dropouts differed in levels of depression and self‐efficacy but not in procrastination or anxiety (see Supporting Information Table S1). In Sample 3, the two groups differed in levels of depression and procrastination but not in self‐efficacy or anxiety (see Supporting Information Table S2).

### 2.2. Procedures

To manage participants effectively and minimize attrition, dedicated WeChat/QQ groups were established prior to data collection, and incentive strategies encouraging participant retention were implemented across the samples. In detail, participants from Sample 1 would receive additional compensation if they completed the daily assessments on time. In Samples 2 and 3, incentives were progressively increased across waves, and eligibility for each subsequent survey was restricted to participants who had completed the preceding wave.

During tracking, all surveys were administered online via reliable platforms (i.e., Wenjuanxing or *Credamo*), with distribution schedules varying across samples. Overall, Sample 1 adopted a daily dual‐administration design aligned with Chinese students’ typical routines, whereas Samples 2 and 3 followed a single‐administration schedule within specific multiday windows. Specifically, in Sample 1, items assessing previous‐day bedtime procrastination and same‐day get‐up procrastination were delivered at 7:00 a.m., while the remaining measures were administered at 10:00 p.m. In contrast, Samples 2 and 3 completed the surveys per wave within designated time windows: (a) Sample 2: November 16–20, 2023 (Wave 1), December 16–20, 2023 (Wave 2), and January 16–20, 2024 (Wave 3); (b) Sample 3: October 14–November 3, 2024 (Wave 1), March 24–April 21, 2025 (Wave 2), and October 9–November 5, 2025 (Wave 3).

Across samples, participants completed the surveys following similar procedures. They provided the informed consents, were free to withdraw at any time, and completed online surveys with standardized instructions emphasizing genuine responses (see Supporting Information for more details). Subsequently, they got paid after the surveys. The study protocol was approved by the Institutional Review Board (IRB) of Zhejiang University (Approval Number: ZJU2024084).

### 2.3. Assessments

All three datasets included measures of procrastination, depression, anxiety, and self‐efficacy, employing daily assessments to capture state levels and additional assessments to assess general tendencies. Specifically, Sample 1 completed daily assessments with items anchored to experiences or behaviors from that same day, whereas Samples 2 and 3 finished measures designed to assess more stable, trait‐like inclinations (see Supporting Information for details).

#### 2.3.1. Depressive Symptoms

A consistent set of nine depressive symptoms was measured across all three samples using the Beck Depression Inventory (BDI) or its revised version (BDI‐II) [20, 21]. Specifically, Samples 1 and 2 completed the original BDI, which assessed symptoms including pessimism, past failure, guilt, self‐dislike, suicidal thoughts, loss of interest, indecisiveness, appetite, and fatigue. Sample 3 completed the BDI‐II, which included the same nine symptoms.

#### 2.3.2. Procrastination

The assessment of procrastination was designed to capture the full spectrum of this construct by measuring it at both specific and general levels across the samples. The task‐specific procrastination was measured in Sample 1 and encompassed three distinct domains: academic procrastination, bedtime procrastination, and get‐up procrastination [22]. Samples 2–3 assessed the general tendency of procrastination using the Pure Procrastination Scale and a short version of Lay’s General Procrastination Scale, both of which contained highly similar items [23, 24].

#### 2.3.3. Anxiety

Anxiety was operationalized as a unified construct across samples, with different instruments employed for its assessment. In detail, Sample 1 assessed state anxiety at the daily level using a single item [25]. Samples 2 and 3, by contrast, indexed general anxiety using multi‐item instruments, namely, the subscale of the Spielberger State‐Trait Anxiety Inventory [26] and the Beck Anxiety Inventory [27], respectively.

#### 2.3.4. Self‐Efficacy

Self‐efficacy was operationalized as a common construct across samples, while the levels of assessment varied. Sample 1 measured state self‐efficacy using a single item, while Sample 2 and 3 completed the General Self‐Efficacy Scale to measure their dispositions of self‐efficacy [28].

### 2.4. Statistical Analyses

#### 2.4.1. Preprocessing of Data

We screened data across each sample based on the following criteria (see Participants for details). First, responses submitted outside the designated time windows were excluded. In Sample 1, morning‐distributed items were required to be completed within the same morning and evening‐distributed items by midnight of the same day. After screening, 2074 valid cases per variable were retained from 2278 morning responses (response rate: 91.04%) and 2427 evening responses (response rate: 85.46%). Similarly, in Samples 2 and 3, responses completed outside the specified time windows were removed (see Designs and Procedures for details). Second, responses were considered invalid if participants provided duplicate answers to items within the same assessment window. Third, participants from Samples 2 and 3, who failed any of the attention checks, were excluded from the analyses. For instance, “For this item, please select ‘fairly consistent’.” Finally, for Sample 3, responses were considered invalid if the completion time was below 360 s or exceeded 2000 s.

Based on the screened data, we further preprocessed these data for enhancing the interpretability of results and excluding between‐subject variance. First, data were standardized within each sample to eliminate scale‐unit differences. Second, data were person‐mean centered by subtracting each individual’s mean score across time from their observed score at each time point, thereby isolating within‐person fluctuations.

#### 2.4.2. Measurement Invariance Tests

To assess whether the scales measured the same construct across different time points, we conducted measurement invariance tests for all scales (Sample 1: depression; Samples 2–3: depression, anxiety, self‐efficacy, procrastination), following the standard sequence of configural, metric, and scalar models (see Supporting Information for details) [29, 30].

Overall, except for the procrastination scale, the depression, anxiety, and self‐efficacy scales might not capture the same underlying psychometric constructs across time. Specifically, results showed strong invariance for procrastination across Samples 2 and 3 (see Supporting Information Tables S4 and S5). By contrast, the scales (i.e., depression, anxiety, and self‐efficacy) showed partial invariance after indices modification across Samples 2 and 3 (see Supporting Information Tables S4 and S5) [31]. Additionally, the basic factor structure (i.e., configural invariance) of the depression scale showed suboptimal model fitness in Sample 1 (TLI = 0.79, RMSEA = 0.13; see Supporting Information Table S3). Together, these findings indicated that analyses based on total scores were appropriate for the procrastination scale, but not for the depression, anxiety, and self‐efficacy scales. Hence, we performed the symptom‐level analyses to probe the temporal associations between depressive symptoms and procrastination.

#### 2.4.3. Testing Timescale‐Dependent Links: Multigroup Structural Equation Modeling (SEM)

This study probed whether the longitudinal associations between procrastination and depressive symptoms varied across short‐term and long‐term time intervals. To address this issue, we applied the SEM analyses to conduct the model comparisons between a constrained model assuming time‐invariant pathways and an unconstrained model permitting time‐variant pathways.

To examine whether the associations between depressive symptoms and procrastination varied across timescales (H_1_) or remained invariant (H_0_), we performed the model comparisons between timescale‐variant and timescale‐invariant models. In detail, a timescale‐variant model allowed the structural parameters (e.g., regressive and autoregressive paths) to vary across different timescales. By contrast, a timescale‐invariant model imposed equality constraints on the regressive paths across timescales. Given that the autoregressive paths were likely to vary across timescales, the timescale‐variant model consequently allowed the autoregressive paths to be freely estimated across different timeframes. The two models were compared using chi‐square difference tests. To provide robust evidence for the strength of both the null hypothesis H_0_ and the alternative hypothesis H_1_, Bayes factors were simultaneously estimated to quantify evidence for H_0_ versus H_1_, with BF_10_ > 3 indicating support for H_1_ and BF_10_ < 1/3 indicating support for H_0_ [32, 33]. Additionally, the directionality between variables was determined based on which model demonstrated better fit in model comparison analyses.

Specifically, SEM analyses were conducted to examine the lagged effects of depressive symptoms on procrastination, as well as its reverse pathway from procrastination to depressive symptoms. First, to construct the predictive role of depressive symptoms on procrastination, we constructed a model by entering nine depressive symptoms (*t* − 1) and sum scores of procrastination (*t* − 1) as predictors and sum scores of procrastination (*t*) as dependent variables. Second, to probe the lagged effects of the reverse pathway (i.e., from procrastination to depressive symptoms), another model was performed by specifying sum scores of procrastination (*t* − 1) and autoregressive paths of depressive symptoms (*t* − 1) as predictors and each depressive symptom (*t*) as dependent variables.

To test the robustness of findings, we conducted another model comparison with a modification: the parameter constraints (e.g., on the autoregressive paths) were altered for both timescale‐invariant and timescale‐variant models. Specifically, unlike the previous models in which the autoregressive paths were freely estimated at each timescale, the current models constrained these paths to be equal across all timescales.

As a whole, other details for the multigroup SEM analyses were listed as below. In detail, timescale (i.e., 1, 30, and 180 days) was specified as the grouping variable, and participants were specified as a clustering variable for accounting for the nonindependence of observations. To further address potential attrition bias, the full information maximum likelihood (FIML) estimation was used to address missing data. Overall, analyses were implemented on the standardized and individual‐centered data by using the R package *lavaan*.

## 3. Results

### 3.1. Descriptive Statistics

Descriptive statistics of the variables across datasets are presented in Supporting Information Tables S6–S8.

### 3.2. Lagged Effects of Depressive Symptoms on Procrastination Across Timescales

Overall, findings indicated the lagged effects of depressive symptoms on procrastination remained consistent across timescales. Specifically, results showed that timescale‐invariant model outperformed the the timescale‐variant model [Δ*χ* ^2^(18) = 13.41, *p* = 0.77; BF_10_ = 0.00; see Table 1]. Additionally, the timescale‐invariant model showed good model fitness [*χ* ^2^(18) = 13.41, CFI = 1.00, TLI = 1.00, RMSEA = 0.00, SRMR = 0.01].

**Table 1:** Model comparisons on predicting procrastination with depressive symptoms.

Models (hypothesis)	Model comparisons		
	BIC	Δ*χ* ^2^ (*p*)	BF_10_
Time‐invariant model (H0)	5871.30	13.41 (0.77)	0.00
Time‐variant model (H1)	6008.70		

Besides, findings suggested that depressive symptoms had negligible effects on procrastination. Results based on the timescale‐invariant model showed that neither of the depressive symptoms predicted subsequent procrastination (see Supporting Information Table S9). To quantify evidence for this null hypothesis, we estimated the Bayes factor to compare a baseline model (Model 0), which included only the autoregressive path of procrastination as a predictor, with a full model (Model 1) that included both depressive symptoms and the autoregressive path of procrastination as predictors. This exploratory finding showed that the model without depressive symptoms outperformed the model with these symptoms (BIC _Model 0_ = 7209.10, BIC _Model 1_ = 7280.30; BF_10_ = 0.00).

Additionally, the robustness check replicated these results. Specifically, a supplementary SEM analysis with alternative parameter constraints on autoregressive paths was employed to compare the timescale‐variant and timescale‐invariant models. The results similarly found no significant predictive effects of depressive symptoms on procrastination (see Supporting Information Tables S10 and S11).

### 3.3. Lagged Effects of Procrastination on Depressive Symptoms Across Timescales

As a whole, the findings implied that the reverse pathway (i.e., from procrastination to each depressive symptom) remained consistent across temporal intervals. In detail, it was found that the timescale‐invariant model outperformed the timescale‐variant model [Δ*χ* ^2^(18) = 24.60, *p* = 0.14; BF_10_ = 0.00; see Table 2]. The timescale‐invariant model showed good model fitness [*χ* ^2^(234) = 314.12, CFI = 0.99, TLI = 0.99, RMSEA = 0.02, SRMR = 0.03].

**Table 2:** Model comparisons on predicting depressive symptoms with procrastination.

Models (hypothesis)	Model comparisons		
	BIC	Δ*χ* ^2^ (*p*)	BF_10_
Time‐invariant model (H0)	65,040	24.60 (0.14)	0.00
Time‐variant model (H1)	65,164		

Furthermore, the findings indicated a directional effect of procrastination on depressive symptoms. Specifically, procrastination was found to longitudinally predict increases in depressive symptoms, including past failure and fatigue (see Table 3).

**Table 3:** The lagged effects of procrastination on nine depressive symptoms.

Pathways	*β*	*z*‐Value	*p*
Procrastination (*t* − 1) → pessimism (*t*)	0.00	0.10	0.92
Procrastination (*t* − 1) → past failure (*t*)	0.04	2.55	0.01 ^∗^
Procrastination (*t* − 1) → guilt (*t*)	0.00	−0.06	0.95
Procrastination (*t* − 1) → self‐dislike (*t*)	0.02	1.10	0.27
Procrastination (*t* − 1) → suicidal thoughts (*t*)	0.01	0.26	0.79
Procrastination (*t* − 1) → loss of interest (*t*)	0.01	0.47	0.64
Procrastination (*t* − 1) → indecisiveness (*t*)	0.02	1.08	0.28
Procrastination (*t* − 1) → appetite (*t*)	0.04	2.02	0.04 ^∗^
Procrastination (*t* − 1) → fatigue (*t*)	0.02	0.77	0.44

*Note:* ^∗^, *p* < 0.05.

In addition, the replication analysis replicated the results mentioned above. In detail, it was found that procrastination positively predicted subsequent depressive symptoms, and that this lagged association was not time‐dependent (see Supporting Information Tables S12 and 13).

## 4. Discussion

The present study examined longitudinal associations between procrastination and depressive symptoms across three temporal intervals (i.e., daily, monthly, and half‐yearly). Across all timescales, depressive symptoms showed negligible predictive power on subsequent procrastination, whereas procrastination consistently predicted increases in depressive symptoms. Notably, this directional pattern remained stable across timescales, indicating that the association between procrastination and depressive symptoms is characterized by directional asymmetry rather than timescale‐specific dynamics. Together, these findings suggest that procrastination may serve as a consistent but weak predictor of depressive symptoms, while depressive symptoms do not appear to be a reliable factor of procrastination within the examined time ranges.

From a dynamic‐systems perspective, vicious‐cycle accounts typically propose that procrastination and negative emotions form a closed, mirror‐symmetric feedback loop, characterized by bidirectional influences of comparable magnitude [6]. The present findings challenge this assumption by demonstrating a clear directional asymmetry: procrastination predicted subsequent depressive symptoms at multiple timescales, whereas depressive symptoms did not reliably predict subsequent procrastination. These results suggest that the interplay between procrastination and depressive symptoms is unlikely to be governed by a single, bidirectionally operating mechanism. Consequently, increases in either procrastination or depressive symptoms do not necessarily give rise to a mutually reinforcing vicious cycle.

This asymmetry also has implications for the emotion‐driven procrastination hypothesis, a key component of the emotion‐regulation account. This hypothesis proposes that task‐elicited negative affect enhances the motivation to disengage from a specific task, thereby predicting subsequent procrastination [10, 34]. However, such longitudinal relationships were not observed when depressive symptoms were examined. Importantly, task‐elicited negative emotions differ from depressive mood in several respects: the former is task‐bound, momentary, and high in arousal, whereas the latter is diffuse, enduring, and low in arousal. Accordingly, the emotion‐driven procrastination hypothesis, if present, is more likely to operate at very short timescales and rely on task‐specific affect rather than enduring mood states such as depressive symptoms [35]. In this sense, the hypothesis may help explain why procrastination occurs in the moment, but not why procrastination persists over time.

The current findings may support one of the preconditions for framing interventions around procrastination as a less stigmatizing approach to alleviating depression, with evidence showing procrastination predicted subsequent depressive symptoms across temporal intervals. Although effective treatments for depression are available, stigma often hinders help‐seeking and treatment adherence [36]. Researchers thus propose that reframing interventions as targeting procrastination may mitigate such barriers, given that procrastination is typically perceived as a behavioral habit rather than a mental health condition [37, 38]. This approach is viable only if procrastination temporally precedes depressive symptoms; otherwise, depressed individuals would have little motivation to participate in “procrastination‐focused” interventions. Our findings provide empirical support for this precondition. Future research could examine whether explicitly framing interventions as targeting procrastination increases engagement willingness compared to traditional depression‐framed approaches.

Rather than reflecting temporally distinct mechanisms, worse‐off effects may arise from stable evaluative systems that operate across timescales. Prior distinctions between self‐evaluative consequences and situational setbacks implicitly suggest that the strength of procrastination‐depression associations should vary with temporal interval. However, the present findings point to an alternative possibility: these processes may be shaped by shared evaluative frameworks that consistently link procrastination to emotional distress over time. One candidate explanation is that broader social evaluative contexts—such as norms emphasizing efficiency, productivity, or meritocratic achievement—simultaneously intensify self‐critical reactions and heighten sensitivity to performance‐related setbacks [39]. Within such frameworks, procrastination may be interpreted as a failure to meet socially valued standards, thereby contributing to depressive symptoms independently of temporal resolution. Future research could test this account by examining whether perceived performance pressure, internalized achievement values, or sociocultural evaluation norms moderate or mediate longitudinal associations between procrastination and depression. Integrating individual, contextual, and cultural‐level measures into longitudinal designs may help clarify whether worse‐off effects reflect distinct temporal mechanisms or shared evaluative systems that operate across time.

Nevertheless, the present study has several methodological limitations. First, the observational longitudinal design precludes strong causal inferences. Although cross‐lagged analyses establish temporal ordering, unmeasured confounding factors—such as stable individual differences in emotion‐regulation capacity—cannot be ruled out [10, 40]. Second, nonrandom attrition may introduce bias into the estimation of longitudinal associations between procrastination and depressive symptoms. Although methods such as FIML and multiple imputation are appropriate under the assumption of missing at random, they cannot fully account for the observed attrition patterns, leaving the possibility of residual bias in the longitudinal estimates. Third, conceptual heterogeneity arising from the use of both the BDI and BDI‐II may introduce bias into the estimation of temporal associations, despite the application of statistical procedures (e.g., standardization and person‐mean centering) to enhance comparability.

Besides, the current work has several limitations that warrant caution in interpreting and generalizing the findings. First, the modest effect sizes observed necessitate careful consideration of their practical significance. Specifically, the effect of procrastination on subsequent depressive symptoms was small (*β* ≈ 0.04), limiting its relevance as a behavioral risk factor for depression. Accordingly, any practical implications—such as assuming that interventions targeting procrastination will yield downstream improvements in depressive symptoms—should be interpreted with caution to avoid overgeneralization. Second, the present results do not directly demonstrate that the observed directional pattern at both the state and trait levels is driven by a common mechanism. For instance, the underlying processes, particularly situational setbacks (e.g., task failures), may unfold over longer time intervals to exert their effects on the observed association, suggesting the possibility of distinct mechanisms that were not captured in this study. Third, the generalizability of the findings may be limited by the sampled population and study contexts. All studies focused on university students, and data collection occurred during specific academic periods. These contextual features may shape both procrastination behavior and emotional experiences, and caution is therefore warranted when extending the present findings to nonstudent populations or broader life contexts.

## 5. Conclusion

In conclusion, the present study revealed a clear directional asymmetry in the longitudinal relationship between depressive symptoms and procrastination. Depressive symptoms showed minimal predictive effects on subsequent procrastination, whereas procrastination consistently predicted higher levels of depressive symptoms across timescales. These findings challenge assumptions of a bidirectional vicious cycle and instead suggest that procrastination may function as a consistent but weak factor of depressive symptoms.

## Funding

This study is funded by the STI 2030—Major Projects (Grant 2021ZD0200400) and the Natural Science Foundation of Zhejiang Province (Grant MS25C090027), the Fundamental Research Funds for the Central Universities (Grant S20230009), the Project supported by the Scientific Research Starting Foundation (Grant 2101074201), and a grant from the Zhejiang Key Laboratory of Neurocognitive Development and Mental Health.

## Ethics Statement

The present study was conducted in line with principles in the Declaration of Helsinki.

## Conflicts of Interest

The authors declare no conflicts of interest.

## Supporting Information

Additional supporting information can be found online in the Supporting Information section.

## Supporting information


**Supporting Information** Additional supporting information includes Tables S1–S13. Table S1: The attrition analyses. Table S2: The attrition analyses. Table S3: The results of the measurement invariance test. Table S4: The model fitness of measurement invariance tests. Table S5: The model fitness of measurement invariance tests. Table S6: The descriptive statistics of variables. Table S7: The descriptive statistics of variables at each wave. Table S8: The descriptive statistics of variables at each wave. Table S9: The lagged effects of nine depressive symptoms in procrastination. Table S10: Model comparisons on predicting procrastination with depressive symptoms. Table S11: The lagged effects of nine depressive symptoms on procrastination. Table S12: Model comparisons on predicting depressive symptoms with procrastination. Table S13: The lagged effects of procrastination on nine depressive symptoms.

## Data Availability

The data can be accessed from https://osf.io/jz9sc/.
