# Supplementary material for: Directional Asymmetry Without Timescale Dependence: Longitudinal Associations Between Procrastination and Depressive Symptoms
Source: Depress Anxiety. 2026 Apr 29;2026:3056943. doi: 10.1155/da/3056943 (PMC13127549; doi:10.1155/da/3056943)
Supplement: Supplementary file 1 — Supporting Information Additional supporting information includes Tables S1–S13. Table S1: The attrition analyses. Table S2: The attrition analyses. Table S3: The results of the measurement invariance test. Table S4: The model fitness of measurement invariance tests. Table S5: The model fitness of measurement invariance tests. Table S6: The descriptive statistics of variables. Table S7: The descriptive statistics of variables at each wave. Table S8: The descriptive statistics of variables at each wave. Table S9: The lagged effects of nine depressive symptoms in procrastination. Table S10: Model comparisons on predicting procrastination with depressive symptoms. Table S11: The lagged effects of nine depressive symptoms on procrastination. Table S12: Model comparisons on predicting depressive symptoms with procrastination. Table S13: The lagged effects of procrastination on nine depressive symptoms. [file DA-2026-3056943-s001.docx]

**Directional Asymmetry Without Timescale Dependence: Longitudinal Associations Between Procrastination and Depressive Symptoms**

**Methods**

**Participants**

In Sample 2, 22.46% (n = 144) and 13.73% (n = 88) of participants experienced severe anxiety (score > 53) and depression (score > 16) at baseline, respectively (Beck & Beck, 1972; Moon et al., 2015). In Sample 3, 20.99% (n = 353) and 3.80% (n = 64) of participants suffered from severe anxiety (score > 36) and depression (score > 29) at baseline, respectively (Beck et al., 1988, 1996).

***Sample 2.*** The attrition analyses showed that compared to completers, dropouts from wave 2 and wave 3 showed significantly higher levels of depression. Dropouts from wave 3 showed significantly higher levels of self-efficacy (see Table S1).

**Table S1. The attrition analyses.**

| **Group** | **Variable** | **Mean (Completer)** | **Mean (Dropout)** | **t** | ***p*** | **Cohens' d** |
| --- | --- | --- | --- | --- | --- | --- |
| Group1_2 | self-efficacy | 24.08 | 25.03 | −1.73 | 0.09 | −0.16 (−0.32, 0.01) |
| Group1_2 | procrastination | 36.36 | 37.29 | −0.93 | 0.36 | −0.08 (−0.24, 0.08) |
| Group1_2 | anxiety | 46.07 | 46.34 | −0.28 | 0.78 | −0.02 (−0.19, 0.14) |
| Group1_2 | depression | 7.69 | 9.81 | −3.46 | 0.00 | −0.31(−0.47, −0.14) |
| Group1_3 | self-efficacy | 23.86 | 25.02 | −2.36 | 0.02 | −0.19 (−0.34, −0.03) |
| Group1_3 | procrastination | 36.21 | 37.20 | −1.07 | 0.29 | −0.09 (−0.24, 0.07) |
| Group1_3 | anxiety | 46.38 | 45.92 | 0.54 | 0.59 | 0.04 (−0.11, 0.20) |
| Group1_3 | depression | 7.56 | 9.36 | −3.26 | 0.00 | −0.26 (−0.42, −0.10) |

Notes: Group 1_2 referred to the attrition analysis comparing completers and Wave 2 dropouts, whereas Group 1_3 referred to the analysis comparing completers and Wave 3 dropouts.

***Sample 3.*** The attrition analyses found that compared to completers, dropouts from wave 2 showed higher levels of depression, and those from wave 3 exhibited higher levels of procrastination (see Table S2).

**Table S2. The attrition analyses.**

| **Group** | **Variable** | **Mean (Completer)** | **Mean (Dropout)** | **t** | ***p*** | **Cohens' d** |
| --- | --- | --- | --- | --- | --- | --- |
| Group1_2 | self-efficacy | 28.56 | 28.61 | −0.20 | 0.84 | −0.01 (−0.11, 0.09) |
| Group1_2 | procrastination | 25.01 | 25.63 | −1.69 | 0.09 | −0.09 (−0.09, 0.01) |
| Group1_2 | anxiety | 29.96 | 30.50 | −0.91 | 0.36 | −0.05 (−0.16, 0.06) |
| Group1_2 | depression | 9.31 | 10.41 | −2.39 | 0.02 | −0.12 (−0.22, −0.02) |
| Group1_3 | self-efficacy | 28.61 | 28.54 | 0.27 | 0.79 | 0.01 (−0.08, 0.11) |
| Group1_3 | procrastination | 24.77 | 25.84 | −2.99 | 0.00 | −0.15 (−0.24, −0.05) |
| Group1_3 | anxiety | 30.07 | 30.22 | −0.26 | 0.80 | −0.01 (−0.12, 0.09) |
| Group1_3 | depression | 9.38 | 10.14 | −1.73 | 0.08 | −0.09 (−0.18, 0.01) |

Notes: Group 1_2 referred to the attrition analysis comparing completers and Wave 2 dropouts, whereas Group 1_3 referred to the analysis comparing completers and Wave 3 dropouts.

**Designs and Procedures**

***Sample 1.*** Before tracking, participants received standardized instructions indicating that “It is common that our thoughts and emotions fluctuate from day to day or remain relatively stable. Please respond to each item based on your genuine thoughts or feelings for that day. It would be better to finish the items promptly upon receiving them. There are no right or wrong answers, and your compensation is not contingent on your responses.”

**Assessments**

***Sample 1.***

Depressive symptoms were assessed using the *Beck Depression Inventory* (BDI) (Beck & Beck, 1972). For example, “Today, I feel pessimistic about what the future holds”. Besides, to capture the typical domains in which procrastination occurred, we used three items to assess real-life procrastination (Kroese et al., 2014): (a) academic procrastination: “Today, I put off my academic plans and obligations”; (b) bedtime procrastination: “When it was time to go to bed last night, I felt sleepy but still did not go to sleep”; (c) get-up procrastination: “Although I was fully awake this morning, I still failed to get out of bed on time”. Self-efficacy was assessed with the item “Right now, I believe I can cope with whatever the future brings”, while anxiety was measured with the item “Today, I feel anxious” (Klimstra et al., 2016). Notably, all the items were rated on a 9-point Likert scale (0, strongly disagree; 8, strongly agree).

***Sample 2****.*

***Depressive symptoms*.** The Beck Depression Inventory (BDI) (Beck & Beck, 1972) was used to assess the depressive symptoms. Using “sense of failure” as an example, the item is phrased as: “I feel like a complete failure.” Participants rated each item on a 4-point Likert scale (0 = absent or mild; 3 = severe). Scores were summed across the 13 items to yield a total score ranging from 0 to 39, with higher scores reflecting greater levels of depressive symptoms. The scale demonstrates strong internal consistency (Cronbach’s α = 0.88) (Leahy, 1992).

***Procrastination***. The Pure Procrastination Scale (PPS) consists of 12 items (Svartdal et al., 2019; Svartdal & Steel, 2017). Subjects responded to the items following a 5-point Likert rating (1, strongly disagree; 5, strongly agree). The scores on all 12 items were summed up with a maximum score of 60. Higher scores indicate higher levels of procrastination in individuals. This scale shows good reliability (Cronbach’s *α* = 0.92) (Steel, 2010).

***General Self-efficacy***. The General Self-Efficacy Scale (GSES) totally consists of 10 items (Schwarzer & Jerusalem, 1995), such as “I can always find a solution to problems when I encounter them”. GSES employed a 4-point Likert scale which ranged from 1 (i.e., completely incorrect) to 4 (i.e., completely correct). We summed up the scores of all 10 items, with a maximum score of 40. Higher scores indicate higher levels of self-efficacy in individuals. This scale shows an adequate internal consistency (Cronbach’s *α* = 0.8) (Schwarzer & Jerusalem, 1995).

***Anxiety***. The subscale of the Spielberger State-Trait Anxiety Inventory (STAI) comprises 20 descriptive items (Spielberger, 1983), such as “I experience such intense feelings of frustration that I cannot get rid of them.” Participants rated each item on a 4-point Likert scale (1 = almost never; 4 = almost always). Scores were summed across the 20 items, yielding a total score ranging from 20 to 80, with higher scores reflecting greater levels of anxiety. The scale demonstrates good reliability (Cronbach’s *α* = 0.89) (Barnes et al., 2002).

***Sample 3.***

***Depressive symptoms*.** The Beck Depression Inventory (BDI-II) was used to measure depressive symptoms (Beck et al., 1996). Taking “past failure” as an example, participants received a score of 0 when they indicated “I don’t think I’m a loser,” and a score of 3 when they indicated “I think I’m a complete loser.” The summed score ranges from 0 to 63, with higher scores indicating greater levels of depression. The scale demonstrates strong internal consistency (Cronbach’s *α* = 0.93) (Beck et al., 1996).

***Procrastination***. The short version of Lay’s General Procrastination Scale (GPS) consists of 9 items (Sirois et al., 2019), such as “I am continually saying I will do it tomorrow”. Subjects responded on a 5-point Likert scale (1, strongly disagree; 5, strongly agree). The scores on all items were summed up with a maximum score of 45. Higher scores indicate higher levels of procrastination. This scale shows good reliability (Cronbach’s α = 0.89) (Sirois et al., 2019).

***Anxiety***. The Beck Anxiety Inventory (BAI) consists of 21 items to measure anxiety (Beck et al., 1988). The items assess numb, feeling hot, wobbliness in legs, unable to relax, fear of the worst happening, dizzy, heart racing, unsteady, terrified, nervous, feeling of choking, hands trembling, shaky, fear of losing control, difficulty breathing, fear of dying, scared, discomfort in abdomen, faint, face flushed, sweating (not due to heat). Participants rated each item on a 4-point Likert scale (0, not at all; 3, severely). The summed score ranges from 0 to 63, with higher scores indicating greater anxious levels. The scale shows good internal consistency (Cronbach's α = 0.92) (Beck et al., 1988).

***General Self-efficacy***. This scale was identical to that used in Sample 2.

**Statistical analyses**

**Measurement invariance tests**

To test the measurement invariance of scales across time, we performed the configural, metric, and scalar models sequentially across three samples. First, a configural invariance model was estimated to examine whether the same latent structure was applicable across three waves. Next, metric invariance was tested by constraining factor loadings to be equal across time, and scalar invariance was tested by additionally constraining item intercepts. All models were estimated using confirmatory factor analysis (CFA) in the R package *lavaan* with the robust maximum likelihood estimator (MLR). The model fitness was evaluated by the ΔCFI < 0.01 and ΔRMSEA < 0.015 (Chen, 2007; Mackinnon et al., 2022). Additionally, based on the attrition analyses, we performed the multiple imputation (m _sample 2_ = 35, m _sample 3_ = 45) using the *mice* package in R (Van Buuren & Groothuis-Oudshoorn, 2011). Thus, the measurement invariance tests were performed across imputed datasets for both Samples 2-3, and the reported fit indices represented averages across these datasets.

***Sample 1***. To reduce the number of estimated parameters, the depressive items were parceled into two subdimensions representing a latent depression construct: (a) affective-cognitive symptoms: mood (BDI1), pessimism (BDI2), sense of failure (BDI3), lack of satisfaction (BDI4), feelings of guilt (BDI5), self-hate (BDI6), self-punitive wishes (BDI7), body image (BDI10); (b) somatic-behavior symptoms: social withdrawal (BDI8), indecisiveness (BDI9), work inhibition (BDI11), fatigability (BDI12), loss of appetite (BDI13). The results of measurement invariance tests were listed in the Table S3.

**Table S3. The results of measurement invariance test.**

| **Indices** | **CFI** | **TLI** | **RMSEA** | **SRMR** | **AIC** | **BIC** | **ΔCFI** | **ΔRMSEA** | **ΔAIC** | **ΔBIC** |
| --- | --- | --- | --- | --- | --- | --- | --- | --- | --- | --- |
| Configural | 0.87 | 0.79 | 0.13 | 0.05 | 10381.79 | 11008.51 | - | - | - | - |
| Metric | 0.86 | 0.79 | 0.13 | 0.24 | 10415.41 | 10957.94 | 0.01 | 0.00 | -33.61 | 50.57 |
| Scalar | 0.86 | 0.79 | 0.13 | 0.24 | 10418.71 | 10958.12 | 0.01 | 0.00 | -36.92 | 50.39 |

***Sample 2.*** The results demonstrated acceptable measurement invariance for procrastination owing to the ΔCFI < 0.01 and ΔRMSEA < 0.015 (see Table S4) (Chen, 2007; Mackinnon et al., 2022). However, the initial tests found depression, self-efficacy, and anxiety presented no robust evidence of invariance. After releasing the residual covariance constraints guided by modification indices (MI > 50), the measurement invariance of these constructs showed acceptable measurement invariance (see Table S4) (Putnick & Bornstein, 2016).

**Table S4. The model fitness of measurement invariance tests.**

| **Variables** | **Indices** | **CFI** | **TLI** | **RMSEA** | **SRMR** | **AIC** | **BIC** | **ΔCFI** | **ΔRMSEA** | **ΔAIC** | **ΔBIC** |
| --- | --- | --- | --- | --- | --- | --- | --- | --- | --- | --- | --- |
| PPS | Configural | 0.86 | 0.85 | 0.08 | 0.19 | 60169.92 | 60772.43 | - | - | - | - |
|  | Metric | 0.89 | 0.88 | 0.07 | 0.11 | 59668.63 | 60159.57 | −0.03 | 0.01 | 501.29 | 612.86 |
|  | Scalar | 0.89 | 0.88 | 0.07 | 0.11 | 59729.09 | 60121.84 | −0.02 | 0.01 | 440.83 | 650.59 |
| BDI | Configural | 0.89 | 0.88 | 0.06 | 0.04 | 41582.52 | 42234.12 | - | - | - | - |
|  | Metric* | 0.93 | 0.92 | 0.05 | 0.87 | 41025.04 | 41605.24 | −0.04 | 0.01 | 557.48 | 628.88 |
|  | Scalar* | 0.92 | 0.91 | 0.05 | 0.87 | 41120.24 | 41593.32 | −0.03 | 0.01 | 462.28 | 640.80 |
| GSES | Configural | 0.91 | 0.90 | 0.06 | 0.04 | 36281.66 | 36785.98 | - | - | - | - |
|  | Metric* | 0.90 | 0.89 | 0.06 | 0.80 | 36389.28 | 36826.66 | 0.01 | 0.00 | −107.62 | −40.68 |
|  | Scalar* | 0.89 | 0.89 | 0.06 | 0.80 | 36452.30 | 36809.35 | 0.02 | 0.00 | −170.64 | −23.37 |
| TA | Configural* | 0.86 | 0.85 | 0.05 | 0.07 | 81911.27 | 83004.72 | - | - | - | - |
|  | Metric* | 0.84 | 0.83 | 0.05 | 0.50 | 82301.03 | 83211.48 | 0.02 | 0.00 | −389.76 | −206.76 |
|  | Scalar* | 0.83 | 0.82 | 0.06 | 0.50 | 82527.27 | 83268.14 | 0.04 | 0.00 | −616.00 | −263.42 |

Notes: PPS, procrastination; BDI, depression; GSES, self-efficacy; TA, anxiety.

* indicated that modification indices were applied at this step by freeing item residual covariances with modification indices (MI) values greater than 50.

***Sample 3.*** The results supported acceptable measurement invariance for procrastination, with ΔCFI approaching 0.01 and ΔRMSEA < 0.015 (see Table S5) (Chen, 2007; Mackinnon et al., 2022). However, the initial tests found depression, self-efficacy, and anxiety presented no robust evidence of strong invariance. After releasing the constraints on residual covariances based on the modification indices (MI > 50), the results found that the scales of depression and self-efficacy showed acceptable measurement invariance. The Beck Anxiety Scale’s indices partially reached the criteria of invariance (see Table S5) (Putnick & Bornstein, 2016).

**Table S5. The model fitness of measurement invariance tests.**

| **Variable** | **Indices** | **CFI** | **TLI** | **RMSEA** | **SRMR** | **AIC** | **BIC** | **ΔCFI** | **ΔRMSEA** | **ΔAIC** | **ΔBIC** |
| --- | --- | --- | --- | --- | --- | --- | --- | --- | --- | --- | --- |
| PPS | Configural | 0.92 | 0.91 | 0.06 | 0.04 | 120483.30 | 121037.00 | - | - | - | - |
|  | Metric | 0.91 | 0.91 | 0.06 | 0.13 | 120626.20 | 121076.70 | 0.01 | 0.00 | −142.90 | −39.70 |
|  | Scalar | 0.91 | 0.90 | 0.06 | 0.13 | 120750.60 | 121114.20 | 0.02 | 0.00 | −267.30 | −77.20 |
| GSES | Configural | 0.94 | 0.93 | 0.05 | 0.03 | 92921.09 | 93534.42 | - | - | - | - |
|  | Metric* | 0.90 | 0.89 | 0.06 | 0.93 | 93606.53 | 94176.44 | 0.03 | −0.01 | −685.44 | −642.02 |
|  | Scalar* | 0.89 | 0.89 | 0.06 | 0.93 | 93794.41 | 94266.63 | 0.04 | −0.01 | −873.32 | −732.21 |
| BDI | Configural | 0.88 | 0.87 | 0.04 | 0.04 | 178239.40 | 179509.50 | - |  | - | - |
|  | Metric* | 0.86 | 0.85 | 0.04 | 1.18 | 178924.00 | 180020.40 | 0.02 | 0.00 | −684.60 | −510.90 |
|  | Scalar* | 0.85 | 0.84 | 0.04 | 1.18 | 179400.70 | 180280.00 | 0.03 | 0.00 | −1161.30 | −770.50 |
| BAI | Configural | 0.88 | 0.88 | 0.04 | 0.04 | 176867.60 | 178137.70 | - | - | - | - |
|  | Metric* | 0.90 | 0.89 | 0.04 | 0.82 | 176143.10 | 177283.00 | −0.01 | 0.00 | 724.50 | 854.70 |
|  | Scalar* | 0.78 | 0.78 | 0.06 | 0.89 | 182182.20 | 182942.10 | 0.10 | −0.02 | −5314.60 | −4804.40 |

Notes: PPS, procrastination; BDI, depression; GSES, self-efficacy; BAI, anxiety.

* indicated that the specific step conducted the modification indices, namely releasing the constraints on residual covariances of the items based on the modification indices (MI> 50).

**Results**

**Descriptive statistics**

Table S6 presented the descriptive statistics of behavioral procrastination, depression, anxiety, and self-efficacy across 14 days in Sample 1 (see Table S6).

**Table S6. The descriptive statistics of variables.**

| **Variables** | **Mean** | **SD** |
| --- | --- | --- |
| Academic procrastination | 2.90 | 2.29 |
| Bedtime procrastination | 3.45 | 2.50 |
| Get-up procrastination | 3.13 | 2.49 |
| Anxiety | 2.24 | 2.03 |
| Self-efficacy | 5.64 | 1.56 |
| Mood | 1.53 | 1.81 |
| Pessimism | 1.36 | 1.70 |
| Sense of failure | 1.47 | 1.82 |
| Lack of satisfaction | 1.86 | 1.97 |
| Guilt | 1.30 | 1.70 |
| Self-hate | 1.43 | 1.77 |
| Self-punitive wishes | 0.45 | 1.03 |
| Social withdrawal | 1.39 | 1.79 |
| Indecisiveness | 1.41 | 1.69 |
| Body image | 1.34 | 1.68 |
| Work inhibition | 1.38 | 1.71 |
| Fatigability | 2.18 | 2.08 |
| Appetite | 1.32 | 1.60 |

Notes: The descriptive statistics were averaged across the 14 days.

Table S7 presented the descriptive statistics of procrastination, depression, anxiety and self-efficacy across three waves in Sample 2.

**Table S7. The descriptive statistics of variables at each wave.**

| **Variables** | **Mean** | **SD** | **Skewness** | **Kurtosis** |
| --- | --- | --- | --- | --- |
| procrastination at T1 | 3.06 | 0.97 | −0.21 | −0.92 |
| procrastination at T2 | 3.10 | 0.83 | −0.29 | −0.70 |
| procrastination at T3 | 3.10 | 0.81 | −0.30 | −0.53 |
| depression at T1 | 0.65 | 0.54 | 0.80 | 0.08 |
| depression at T2 | 0.63 | 0.46 | 0.61 | −0.23 |
| depression at T3 | 0.64 | 0.43 | 0.57 | −0.03 |
| anxiety at T1 | 2.31 | 0.54 | 0.10 | −0.36 |
| anxiety at T2 | 2.34 | 0.46 | 0.11 | 0.00 |
| anxiety at T3 | 2.34 | 0.44 | 0.13 | 0.10 |
| self-efficacy at T1 | 2.44 | 0.62 | 0.11 | −0.43 |
| self-efficacy at T2 | 2.41 | 0.51 | 0.17 | 0.14 |
| self-efficacy at T3 | 2.41 | 0.50 | 0.23 | 0.18 |

Notes: Descriptive statistics for all variables were calculated separately within each imputed dataset and subsequently aggregated by averaging across the multiple imputations (m= 35). Variable scores were derived from the mean scores across all items within each respective scale.

T1: early of a semester; T2: middle of a semester; T3: end of a semester.

Table S8 presented the descriptive statistics of procrastination, depression, anxiety and self-efficacy across three waves in Sample 3.

**Table S8. The descriptive statistics of variables at each wave.**

| **variable** | **Mean** | **SD** | **Skewness** | **Kurtosis** |
| --- | --- | --- | --- | --- |
| procrastination at T1 | 2.80 | 0.81 | 0.04 | −0.61 |
| procrastination at T2 | 2.91 | 0.72 | −0.06 | −0.24 |
| procrastination at T3 | 2.90 | 0.71 | −0.10 | −0.04 |
| depression at T1 | 0.46 | 0.42 | 1.29 | 1.58 |
| depression at T2 | 0.43 | 0.37 | 1.44 | 2.82 |
| depression at T3 | 0.42 | 0.36 | 1.54 | 3.65 |
| anxiety at T1 | 1.43 | 0.48 | 1.46 | 1.76 |
| anxiety at T2 | 1.42 | 0.43 | 1.65 | 3.03 |
| anxiety at T3 | 1.42 | 0.42 | 1.73 | 3.33 |
| self-efficacy at T1 | 2.86 | 0.55 | −0.03 | −0.24 |
| self-efficacy at T2 | 2.87 | 0.47 | −0.15 | 0.31 |
| self-efficacy at T3 | 2.88 | 0.45 | −0.25 | 0.83 |

Notes: Descriptive statistics were computed within each imputed dataset (m= 45) and pooled by averaging across imputations. Scale scores were calculated as the mean of all items within each measure.

**Lagged effects of depressive symptoms on procrastination across timescales**

Table S9 presented the lagged effects of depressive symptoms in procrastination.

**Table S9. The lagged effects of nine depressive symptoms in procrastination.**

| **Pathways** | **β** | **z-value** | ***p*** |
| --- | --- | --- | --- |
| Pessimism (t−1) → Procrastination (t) | 0.02 | 1.43 | 0.15 |
| Past failure (t−1) → Procrastination (t) | −0.01 | −0.90 | 0.37 |
| Guilt (t−1) → Procrastination (t) | 0 | 0.04 | 0.97 |
| Self-dislike (t−1) → Procrastination (t) | 0.02 | 1.11 | 0.27 |
| Suicidal thoughts (t−1) → Procrastination (t) | 0.00 | 0.12 | 0.91 |
| Loss of interest (t−1) → Procrastination (t) | 0.00 | −0.26 | 0.80 |
| Indecisiveness (t−1) → Procrastination (t) | 0.01 | 0.61 | 0.54 |
| Appetite (t−1)→ Procrastination (t) | 0.00 | −0.10 | 0.92 |
| Fatigue (t−1)→ Procrastination (t) | 0.01 | −0.74 | 0.46 |

To assess the robustness of our primary findings, we performed a supplementary SEM analysis. This robustness check involved specifying the constrained and unconstrained models with alternative parameter constraints on the autoregressive paths than those used in the main analysis (see Methods in main text for more details).

Overall, the findings indicated the predictions of depressive symptoms on procrastination remained consistent across temporal scale, which was same as those reported in the main text. Specifically, the results showed that the timescale-invariant model outperformed the timescale-variant model [Δχ²(18) = 23.52, *p* = 0.17; BF_10_ = 0.00; see Table S10]. The timescale-invariant model showed marginally acceptable model fit [χ²(20) = 306.40, CFI = 0.53, TLI = 0.30, RMSEA = 0.10, SRMR = 0.03].

In addition, the results based on the timescale-invariant model showed no predictions of depressive symptoms on procrastination (see Table S11). The Bayes factor further provided evidence for this null hypothesis (see details in the Main text).

**Table S10. Model comparisons on predicting procrastination with depressive symptoms.**

| **Models (Hypothesis)** | **Model comparisons** | | |
| --- | --- | --- | --- |
|  | **BIC** | **Δχ² (*p*)** | **BF_10_** |
| Time-invariant Model (H0) | 6250.80 | 23.52 (0.17) | 0.00 |
| Time-variant Model (H1) | 6374.40 |  |  |

**Table S11. The lagged effects of nine depressive symptoms on procrastination.**

| **Pathways** | **β** | **z-value** | ***p*** |
| --- | --- | --- | --- |
| Pessimism (t−1) → Procrastination (t) | 0.02 | 1.22 | 0.22 |
| Past failure (t−1) → Procrastination (t) | -0.01 | -0.35 | 0.73 |
| Guilt (t−1) → Procrastination (t) | 0.00 | -0.29 | 0.77 |
| Self-dislike (t−1) → Procrastination (t) | 0.01 | 0.68 | 0.50 |
| Suicidal thoughts (t−1) → Procrastination (t) | 0.00 | -0.09 | 0.93 |
| Loss of interest (t−1) → Procrastination (t) | 0.00 | -0.33 | 0.74 |
| Indecisiveness (t−1) → Procrastination (t) | 0.01 | 0.65 | 0.52 |
| Appetite (t−1)→ Procrastination (t) | -0.01 | -0.54 | 0.59 |
| Fatigue (t−1)→ Procrastination (t) | -0.01 | -0.54 | 0.59 |

**Lagged effects of procrastination on depressive symptoms across timescales**

Same as those reported in the main text, findings indicated that the lagged effects of procrastination on depressive symptoms remained same across timescales. The results showed that the timescale-invariant model outperformed the timescale-variant model [Δχ²(18) = 36.25, *p* < 0.01; BF_10_ = 0.00; see Table S12]. The timescale-invariant model showed fine model fit [χ²(252) = 2041.39, CFI = 0.82, TLI = 0.72, RMSEA = 0.08, SRMR = 0.08].

Besides, the results based on timescale-invariant model showed that procrastination positively predicted increases in subsequent depressive symptom, especially past failure (see Table S13).

**Table S12. Model comparisons on predicting depressive symptoms with procrastination.**

| **Models (Hypothesis)** | **Model comparisons** | | |
| --- | --- | --- | --- |
|  | **BIC** | **Δχ² (*p*)** | **BF_10_** |
| Time-invariant Model (H0) | 67563 | 36.25 (0.01) | 0.00 |
| Time-variant Model (H1) | 67672 |  |  |

**Table S13. The lagged effects of procrastination on nine depressive symptoms.**

| **Pathways** | **β** | **z-value** | ***p*** |
| --- | --- | --- | --- |
| Procrastination (t−1) → Pessimism (t) | 0.00 | -0.25 | 0.81 |
| Procrastination (t−1) → Past failure (t) | 0.04 | 2.19 | 0.03 * |
| Procrastination (t−1) → Guilt (t) | -0.01 | -0.36 | 0.72 |
| Procrastination (t−1) → Self-dislike (t) | 0.01 | 0.42 | 0.68 |
| Procrastination (t−1) → Suicidal thoughts (t) | 0.00 | -0.02 | 0.99 |
| Procrastination (t−1) → Loss of interest (t) | 0.00 | 0.20 | 0.85 |
| Procrastination (t−1) → Indecisiveness (t) | 0.02 | 0.87 | 0.38 |
| Procrastination (t−1) → Appetite (t) | 0.03 | 1.56 | 0.12 |
| Procrastination (t−1) → Fatigue (t) | 0.01 | 0.66 | 0.51 |

Notes: *, *p* < 0.05.

**References**

Barnes, L. L., Harp, D., & Jung, W. S. (2002). Reliability generalization of scores on the Spielberger state-trait anxiety inventory. *Educational and Psychological Measurement*, *62*(4), 603–618. DOI: https://doi.org/10.1177/0013164402062004005

Beck, A. T., & Beck, R. W. (1972). Screening depressed patients in family practice: A rapid technic. *Postgraduate Medicine*, *52*(6), 81–85. DOI: https://doi.org/10.1080/00325481.1972.11713319

Beck, A. T., Epstein, N., Brown, G., & Steer, R. A. (1988). An inventory for measuring clinical anxiety: Psychometric properties. *Journal of Consulting and Clinical Psychology*, *56*(6), 893. DOI: https://doi.org/10.1037/0022-006X.56.6.893

Beck, A. T., Steer, R. A., & Brown, G. (1996). Beck depression inventory–II. *Psychological Assessment*. DOI: https://doi.org/10.1037/t00742-000

Chen, F. F. (2007). Sensitivity of goodness of fit indexes to lack of measurement invariance. *Structural Equation Modeling: A Multidisciplinary Journal*, *14*(3), 464–504. DOI: https://doi.org/10.1080/10705510701301834

Klimstra, T. A., Kuppens, P., Luyckx, K., Branje, S., Hale III, W. W., Oosterwegel, A., Koot, H. M., & Meeus, W. H. (2016). Daily dynamics of adolescent mood and identity. *Journal of Research on Adolescence*, *26*(3), 459–473. DOI: https://doi.org/10.1111/jora.12205

Kroese, F. M., De Ridder, D. T., Evers, C., & Adriaanse, M. A. (2014). Bedtime procrastination: Introducing a new area of procrastination. *Frontiers in Psychology*, *5*, 89333. DOI: https://doi.org/10.3389/fpsyg.2014.00611

Leahy, J. M. (1992). Validity and reliability of the Beck Depression Inventory‐Short Form in a group of adult bereaved females. *Journal of Clinical Psychology*, *48*(1), 64–68. DOI: [https://doi.org/10.1002/1097-4679(199201)48:1<64::AID-JCLP2270480109>3.0.CO;2-9](https://doi.org/10.1002/1097-4679(199201)48:1%3C64::AID-JCLP2270480109%3E3.0.CO;2-9)

Mackinnon, S., Curtis, R., & O’Connor, R. (2022). A tutorial in longitudinal measurement invariance and cross-lagged panel models using lavaan. *Meta-Psychology*, *6*. DOI: https://doi.org/10.15626/MP.2020.2595

Moon, C., Kang, H., & Jeong, G. (2015). Metabolic change in the right dorsolateral prefrontal cortex and its correlation with symptom severity in patients with generalized anxiety disorder: Proton magnetic resonance spectroscopy at 3 T esla. *Psychiatry and Clinical Neurosciences*, *69*(7), 422–430. DOI:10.1111/pcn.12279

Putnick, D. L., & Bornstein, M. H. (2016). Measurement invariance conventions and reporting: The state of the art and future directions for psychological research. *Developmental Review*, *41*, 71–90. DOI: https://doi.org/10.1016/j.dr.2016.06.004

Schwarzer, R., & Jerusalem, M. (1995). Generalized self-efficacy scale. *J. Weinman, S. Wright, & M. Johnston, Measures in Health Psychology: A User’s Portfolio. Causal and Control Beliefs*, *35*(37), 82–003. DOI:

Sirois, F. M., Yang, S., & van Eerde, W. (2019). Development and validation of the General Procrastination Scale (GPS-9): A short and reliable measure of trait procrastination. *Personality and Individual Differences*, *146*, 26–33. DOI: https://doi.org/10.1016/j.paid.2019.03.039

Spielberger, C. D. (1983). *State-trait anxiety inventory for adults*. DOI: https://doi.org/10.1037/t06496-000

Steel, P. (2010). Arousal, avoidant and decisional procrastinators: Do they exist? *Personality and Individual Differences*, *48*(8), 926–934. DOI:10.1016/j.paid.2010.02.025

Svartdal, F., Klingsieck, K., Steel, P., & Gamst-Klaussen, T. (2019). *Measuring implemental delay in procrastination: Separating onset and sustained goal striving*. DOI: <https://doi.org/10.1016/j.paid.2019.109762>

Svartdal, F., & Steel, P. (2017). Irrational delay revisited: Examining five procrastination scales in a global sample. *Frontiers in Psychology*, *8*, 1927. DOI: https://doi.org/10.3389/fpsyg.2017.01927

Van Buuren, S., & Groothuis-Oudshoorn, K. (2011). mice: Multivariate imputation by chained equations in R. *Journal of Statistical Software*, *45*, 1–67. DOI: https://doi.org/10.18637/jss.v045.i03
